# How to Thread a Needle

**Published:** 1899-09-23

**Authors:** 


### HOW TO THREAD A NEEDLE.

We extract the following from the New York *Medical Record*: "To thread a needle hold it with the ring and little fingers of the left hand, instead of with the thumb and forefinger as is the usual way. This method, according to Dr. Joseph M. Jackson, leaves the thumb and forefinger free to grasp the smallest bit of silk or other suture material as it passes through the eye, and pull it to a safe distance on the other side. This does away with the slipping back, so common in the old way of changing hands, and incidentally decreases profanity." We do not remember to have come across this suggestion before. Certainly it acts very well, although personally we find that we get a firmer grip of the needle by holding it between the middle and ring fingers than between the ring and little fingers. We fancy, however, that it will be found that when the fingers are dry the ordinary housewife's method of holding the needle eye nearly close to the thumb, then closing the finger and thumb upon the protruding point of thread, and then by a rolling action setting the needle free, will not readily be beaten. It is merely a matter of practice. But with wet fingers and wet silk, as in an operation, it is another matter—then, the above suggestion will probably be found useful.

